# A miRNA signature predicts benefit from addition of hypoxia-modifying therapy to radiation treatment in invasive bladder cancer

**DOI:** 10.1038/s41416-021-01326-9

**Published:** 2021-04-12

**Authors:** Mairah T. Khan, Joely J. Irlam-Jones, Ronnie Rodrigues Pereira, Brian Lane, Helen R. Valentine, Kai Aragaki, Lars Dyrskjøt, David J. McConkey, Peter J. Hoskin, Ananya Choudhury, Catharine M. L. West

**Affiliations:** 1 Translational Radiobiology Group, Division of Cancer Sciences, University of Manchester, Manchester Academic Health Science Centre, Christie NHS Foundation Trust Hospital, Manchester, UK; 2 Translational Oncogenomics, Cancer Research UK Manchester Institute, Oglesby Cancer Research Building, University of Manchester, Manchester, UK; 3 Greenberg Bladder Cancer Institute, Johns Hopkins University, Baltimore, MD USA; 4 Department of Molecular Medicine, Aarhus University Hospital, Aarhus, Denmark; 5 Department of Clinical Medicine, Aarhus University, Aarhus, Denmark

**Keywords:** Cancer, Bladder cancer

## Abstract

**Background:**

miRNAs are promising biomarkers in oncology as their small size makes them less susceptible to degradation than mRNA in FFPE tissue. We aimed to derive a hypoxia-associated miRNA signature for bladder cancer.

**Methods:**

Taqman miRNA array cards identified miRNA seed genes induced under hypoxia in bladder cancer cell lines. A signature was derived using feature selection methods in a TCGA BLCA training data set. miRNA expression data were generated for 190 tumours from the BCON Phase 3 trial and used for independent validation.

**Results:**

A 14-miRNA hypoxia signature was derived, which was prognostic for poorer overall survival in the TCGA BLCA cohort (*n* = 403, *p* = 0.001). Univariable analysis showed that the miRNA signature predicted an overall survival benefit from having carbogen–nicotinamide with radiotherapy (HR = 0.30, 95% CI 0.094–0.95, *p* = 0.030) and performed similarly to a 24-gene mRNA signature (HR = 0.47, 95% CI 0.24–0.92, *p* = 0.025). Combining the signatures improved performance (HR = 0.26, 95% CI 0.08–0.82, *p* = 0.014) with borderline significance for an interaction test (*p* = 0.065). The interaction test was significant for local relapse-free survival LRFS (*p* = 0.033).

**Conclusion:**

A 14-miRNA hypoxia signature can be used with an mRNA hypoxia signature to identify bladder cancer patients benefitting most from having carbogen and nicotinamide with radiotherapy.

## Background

A treatment for locally advanced muscle invasive bladder cancer (MIBC) patients is bladder preservation trimodality therapy involving complete transurethral resection of bladder tumour (TURBT) followed by radiotherapy (RT) with a radiosensitiser.^1,2^ The largest trials investigating the potential of combining RT with radiosensitisers were the Bladder Carbogen Nicotinamide (BCON) and the Bladder Cancer 2001 (BC2001) trials.^1^ The BCON trial randomised patients to RT alone or with hypoxia-modifying carbogen and nicotinamide (RT+CON) and showed that the combined treatment improved the 3-year overall survival rate by 13%.^1,3^ BC2001 randomised patients to RT alone or with chemotherapy (5 fluorouracil and mitomycin C) and showed that the combined treatment improved the 2-year locoregional disease-free survival rate by 13%.^1,4^ Different radiosensitising approaches are used, and as high levels of tumour hypoxia are associated with a poor prognosis irrespective of the treatment approach,^5^ a predictive hypoxia biomarker would help select patients for RT with hypoxia-modifying therapy rather than chemo-radiotherapy.^2,6^ Work from our group showed that necrosis, CA9, hypoxia-inducible factor (HIF)-1α and a 24-gene mRNA signature predict benefit for giving CON with RT in BCON.^3,7–9^ The presence of tumour hypoxia is an adverse prognostic feature irrespective of treatment, and we also showed that high 24-gene signature scores associated with a poor prognosis in patients with MIBC who did not receive RT.^9^

microRNAs (miRNAs) are 20–23 bp long single-stranded RNA gene regulators,^10,11^ which have been validated as diagnostic and prognostic biomarkers in oncology.^12–16^ miRNAs are more stable than mRNA in formalin-fixed paraffin-embedded (FFPE) samples due to their small size, making miRNA expression levels in FFPE samples easier to detect.^17^ miRNA hypoxia signatures have not been developed for bladder cancer. Six miRNAs identified in hypoxia experiments in bladder cancer cell lines were reported to have potential as a non-muscle invasive bladder cancer (NMIBC) hypoxia-associated miRNA signature.^18^ These six miRNAs were identified through experiments in cell lines, but no association with prognosis or prediction in clinical data sets was shown.^18^ Among other cancer types, a three-miRNA hypoxia-related signature was recently shown to be prognostic in colorectal cancer.^19^ Given the potential of miRNAs as biomarkers in FFPE samples, we aimed to develop a miRNA hypoxia signature in MIBC and compare its performance to our previously developed 24-gene mRNA signature.^9^

## Methods

### Cell culture and hypoxia experiments

Bladder cancer cell lines (T24, UMUC3, RT4, J82) were purchased from the American Type Culture Collection (Manassas, VA, USA). Cells were grown in RPMI-1640 with L-glutamine (Thermo Fisher Scientific, Paisley, UK) supplemented with 10% foetal calf serum (Sigma-Aldrich, Dorset, UK). Cell lines were authenticated using the Promega Powerplex 21 system (Chilworth, UK) and tested for mycoplasma at the Molecular Biology Core Facility, CRUK Manchester Institute. Cells were seeded in 75-cm^2^ flasks to obtain 60% confluency after 48 h under normoxia. After 24 h in normoxia_,_ media were changed and cells cultured in parallel in normoxia and hypoxia (0.2% and 1% O_2_) for a further 24 h. Hypoxia was obtained using the Whitley H35 Hypoxystation (Don Whitley Scientific Limited, Bingley, UK). Experiments were carried out in biological triplicate.

### Identification of differentially expressed miRNAs

Taqman miRNA array A cards (Thermo Fisher Scientific) were used to identify miRNAs. The array A cards cover 384 well-characterised miRNAs along with negative and endogenous controls (miRBase version 22). Data were generated for all four cell lines grown in 0.2% O_2_ and 21% O_2_ and for the three MIBC cell lines (T24, J82, UMUC3) grown in 1% O_2_.

RNA was extracted using the miRvana RNA Isolation Kit (Thermo Fisher Scientific) and cleaned using the Norgen RNA concentration and clean-up kit (Norgen Biotek Corp, Elmhurst, UK) following the manufacturer’s instructions. In all, 100 ng of RNA from each sample was reverse transcribed using the miRNA reverse transcription kit and primers specific for the array cards as per the manufacturer’s instructions. The cards were run on a Quantstudio 12K (Applied Biosystems, Foster, CA, USA) and analysed with a normalisation threshold (*R*_n_) of 0.2. Ct value ≥30 across all three normoxia and hypoxia triplicates were excluded from analysis as recommended.

Data were normalised using the geometric mean of the array card endogenous controls (RNU48, RNU44, U6snRNA) or occasionally the geometric mean of one or two controls if that increased the stability of values across the samples. Data were analysed using the ΔΔCt method with mean fold changes (2^(−ΔΔCt)) calculated. *p* Values were obtained using an unpaired *t* test with Welch correction as used previously in other studies.^20,21^ Differentially expressed miRNAs were identified as having *p* values <0.05 (unpaired *t* test with Welch correction) and fold changes >1.0. A false discovery rate (FDR) cut-off was considered too stringent for the analysis due to the high intra-group variation and hence was not applied.

### Development of the miRNA signature

miRNAs induced (i.e. upregulated) with fold change >1.0 under 0.2% hypoxia in ≥2 bladder cancer cell lines were used as seed genes to derive a signature. The Cancer Genome Atlas (TCGA) bladder cancer (BLCA) mature miRNA-seq counts (*n* = 409) were downloaded from GDCRNATools^22^ and normalised to reads per million mapped (RPM) counts. TCGA BLCA mRNA data—RNA-Seq by Expectation-Maximisation (RSEM) normalised counts (*n* = 408)—were downloaded from Firebrowse (http://firebrowse.org). RPM and RSEM values with a pseudo-count of 1 added were log_2_ transformed. There were 405 TCGA BLCA patients who had both miRNA-seq and mRNA-seq data. The 405 TCGA BLCA patients were split 70:30 into the training and test cohorts balanced for the proportion of hypoxia classifications (Supplementary Table 1). Overall survival (OS) data were available for 403 of the 405 patients and progression-free survival (PFS) data were available for 404 of the 405 patients.

The Boruta algorithm^23^ was used in the training data set to identify miRNAs important in predicting hypoxic and normoxic tumours defined using the Winter 99-gene hypoxia signature^24^ (Supplementary Methods). Spearman correlations were calculated between the expression levels of the miRNAs identified and Winter signature scores and those significant were selected for the miRNA signature. miRNA signature scores were calculated as mean (miRNAs positively correlated with the Winter hypoxia scores) − mean (miRNAs negatively correlated with the Winter hypoxia scores).

The online tool miRWalk base 2.0 was used to select miRNA targets predicted by three target prediction databases: miRWalk, TargetScan, and miRDB.^25^ Gene enrichment analysis was carried out on the predicted targets in the MiRWalk database.

### miRNA expression cohorts

miRNA-seq (GSE84525) and mRNA (GSE48075) data were available for 62 MIBC patients generated from fresh frozen bladder tumour samples obtained during TURBT.^26,27^ miRNA microarray expression data were also available for 106 bladder cancer patients (79 NMIBC, 27 MIBC) generated from samples acquired during surgery.^28^ No material was shared between institutions. Expression data for 829 miRNAs were generated for 192 patients randomised in the BCON trial (96 RT and 96 RT+CON) using the NanoString NCounter miRNA assay (NanoString Technologies, Seattle, WA, USA) according to the manufacturer’s instructions. Two samples were excluded as they did not pass all the quality control metrics. Further details on the processing of the NanoString generated data are available in the Supplementary Methods. Log_2_-transformed normalised counts of 190 samples in the BCON cohort were used for analyses. The clinico-pathologic details of the 190 BCON patients are shown in Supplementary Table 2.

### Statistical analysis

The Cox regression model was used for univariable and multivariable analyses and to generate hazard ratios (HRs) and 95% confidence intervals (CIs). Log-rank test and Wald statistic compared differences in univariable and multivariable analyses, respectively. Variables with *p* value <0.1 were used in the multivariable analysis models. Data were censored at 5 years. The R package survival (v 3.1–12) was used for Cox analyses.^29^ The R package survminer (v 0.4.6) was used to plot Kaplan–Meier curves.^30^ Data analyses were carried out using R version 3.6.1 (R core team, Vienna, Austria) or GraphPad Prism 7 (San Diego, CA, USA).

Immunohistochemistry generated CAIX, Glut-1 and HIF-1α protein expression data were available for the BCON cohort from a previous study.^8^ The non-parametric Mann-Whitney U test was used to compare median expression levels of CAIX, Glut-1 and HIF-1α expression data in the miRNA hypoxia signature high and low groups.

## Results

### Differential expression analysis

Previously used approaches for developing hypoxia signatures involved using well-known hypoxia-induced genes (seed genes) from cell lines.^31–34^ A similar approach was used for the development of the miRNA hypoxia signature. Candidate seed miRNAs were identified based on differential expression analysis of cell lines cultured under normoxia and hypoxia. Prostate, sarcoma and lung signatures were derived after identifying seed genes using cell lines exposed to 1% O_2_.^33,34^ However, 0.2% O_2_, which is radiobiologically relevant, was used to select seed genes by others.^32,35^

An initial comparison compared miRNAs induced in the three MIBC cell lines (T24, J82 and UMUC3) exposed to 0.2% and 1% O_2_. There were very few differentially expressed miRNAs in 1% O_2_ versus 0.2% O_2_ in the MIBC cell lines (Supplementary Table 3). Hence, for the fourth NMIBC cell line (RT4), array cards were only run for cells grown in 0.2% O_2_. Supplementary Table 3 shows that 41–64 miRNAs were induced at 0.2% O_2_ in the cell lines. The numbers of miRNAs induced at 0.2% O_2_ in at least 2, 3 and 4 cell lines were 62, 26 and 3. miR-210-3p, miR-15b-5p and miR-99b-5p were induced in all the four cell lines. The 62 miRNAs induced in ≥2 cell lines were taken forward as seed genes (Supplementary Table 4).

### Development and validation of a miRNA hypoxia signature

**Fig. 1:**
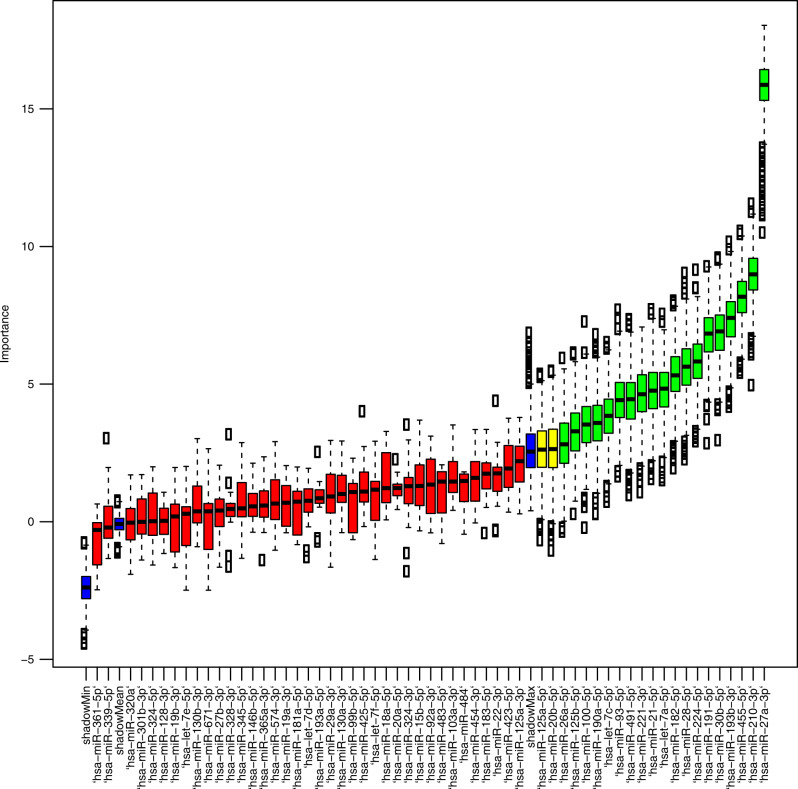
Boruta plot showing that 19 of the 59 miRNA seed genes are important in predicting the Winter mRNA signature classification of hypoxia and normoxia in TCGA BLCA samples. Importance is represented by *Z*-scores. Blue colour boxplots represent *Z*-scores for the maximum, mean and minimum shadow features. The boxplots in green indicate features (miRNAs) classified as important. The features in red were classified as unimportant and the important of the features in yellow could not be determined at the end of 1000 iterations. Importance classification is relative to the shadowMax. TCGA BLCA The Cancer Genome Atlas bladder cancer.

TCGA was used to develop a miRNA signature as it has the only large BLCA cohort with both miRNA and mRNA data. Hypoxia-associated miRNAs were previously identified by using mRNA signatures to classify hypoxic tumours.^36,37^ Therefore, the same approach was used here to filter the in vitro hypoxia-associated miRNAs and identify those most relevant in terms of their ability to classify patient tumours as hypoxic. We considered using the Winter, Buffa, Lendahl and Chi mRNA signatures.^24,31,38,39^ Our 24-gene bladder mRNA hypoxia signature was not used as it was trained on prognosis in the TCGA BLCA data set,^9^ and would classify miRNAs associated with not only hypoxia but also a poor prognosis in our TCGA training data set. The Winter signature was selected as it was the only one of the four signatures that was prognostic for OS in the TCGA BLCA data set (HR = 1.50, 95% CI 1.11–2.03, *p* = 0.008). The Winter signature is not a bladder-specific signature, being neither predictive nor prognostic in BCON,^9^ but it was chosen as it should identify the bladder-specific miRNAs associated with hypoxia both in in vitro and patient samples. Before applying a feature selection method to select the most robust miRNAs for classification, 3 (miR-18b-5p, miR-579-3p, miR-597-5p) of our 62 seed miRNAs were excluded based on having no/low expression across the TCGA BLCA samples. Applying the Boruta algorithm to the TCGA BLCA training data set (*n* = 284) identified 19 of the 59 seed miRNAs as important in predicting the Winter signature classification of hypoxia (Fig. 1). Fourteen of the 19 miRNAs correlated significantly with Winter hypoxia scores (Table 1) and were taken forward for validation as a signature. These 14 miRNAs were in the top 20 based on Gini importance ranking of the 59 miRNAs.

**Table 1:** 14 miRNAs with significant correlations with Winter hypoxia scores.

miRNA	* ρ*	FDR
miR-27a-3p	0.53	0.0
miR-193b-3p	0.35	1.82e−08
miR-455-5p	0.33	6.57e−08
miR-221-3p	0.33	7.39e−08
miR-210-3p	0.30	9.35e−08
miR-21-5p	0.24	1.00e−06
miR-224-5p	0.24	8.84e−05
miR-491-5p	−0.20	1.21e−03
miR-93-5p	−0.20	6.63e−04
miR-182-5p	−0.21	4.50e−04
miR-30b-5p	−0.26	1.43e−05
miR-190a-5p	−0.29	1.56e−06
miR-28-5p	−0.29	1.56e−06
miR-191-5p	−0.29	1.56e−06

*FDR* false discovery rate.

**Fig. 2:**
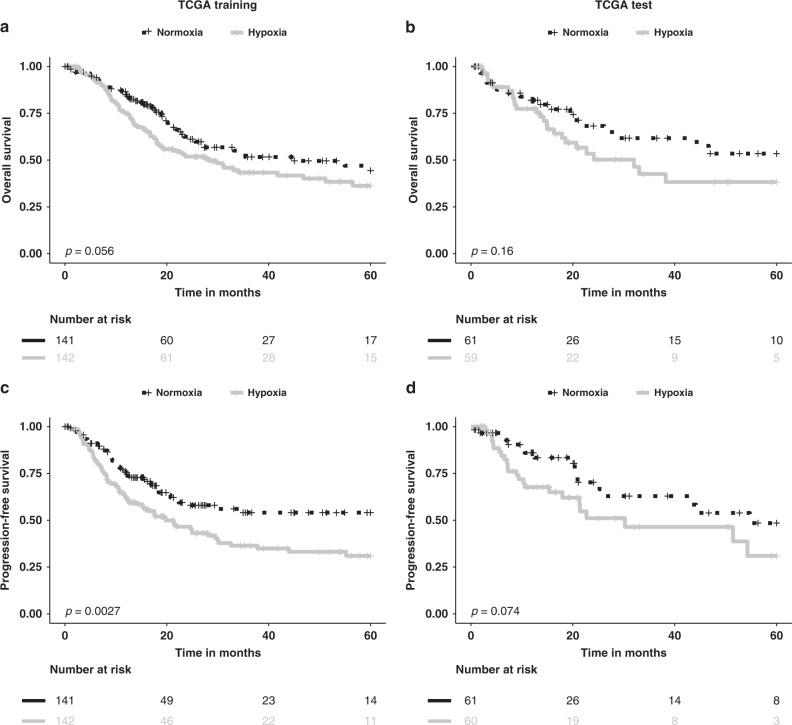
Performance of a 14-miRNA signature in the training and test datasets with stratification by median signature scores. Kaplan–Meier curves for overall survival in the training (**a**) and test (**b**) data sets and for progression-free survival in the training (**c**) and test (**d**) data sets. Patients were stratified by the median of the 14-miRNA signature score. TCGA BLCA The Cancer Genome Atlas bladder cancer.

Stratifying the 14 miRNA signature by quartiles initially showed a median cut-off worked the best for PFS (HR = 1.71, 95% CI 1.20–2.45, *p* = 0.0027) and OS (HR = 1.41, 95% CI 0.99–2.02, *p* = 0.056) in the training data set (Fig. 2a, c and Supplementary Table 5). Similar trends were seen in multivariable analyses (Supplementary Table 5). In the TCGA BLCA test data set (*n* = 121), the miRNA signature showed a trend for PFS (HR = 1.75, 95% CI 0.94–3.27, *p* = 0.074) but was not prognostic for OS (HR = 1.53, 95% CI 0.84–2.76, *p* = 0.16) (Fig. 2b, d and Supplementary Table 6). In the multivariable analyses, the miRNA signature showed a trend for OS; PFS was not tested as none of the clinical variables had *p* values <0.1 (Supplementary Table 6).

### Validation in external cohorts

Prognostic significance of the 14-miRNA signature was explored in a cohort of 62 MIBC patients. The signature was prognostic for OS (HR = 2.56, 95% CI 1.19–5.48, *p* = 0.01) and disease-specific survival (HR = 2.57, 95% CI 1.15–5.73, *p* = 0.02). Multivariable analyses were not significant (Supplementary Table 7). Use of an upper quartile for stratification improved the performance of the signature (Supplementary Fig. 1). For comparison, the results of analyses of the Yang mRNA hypoxia signature are shown in Supplementary Fig. 1. The Yang signature gave prognostic significance only when stratified by the lower quartile (Supplementary Table 7).

**Fig. 3:**
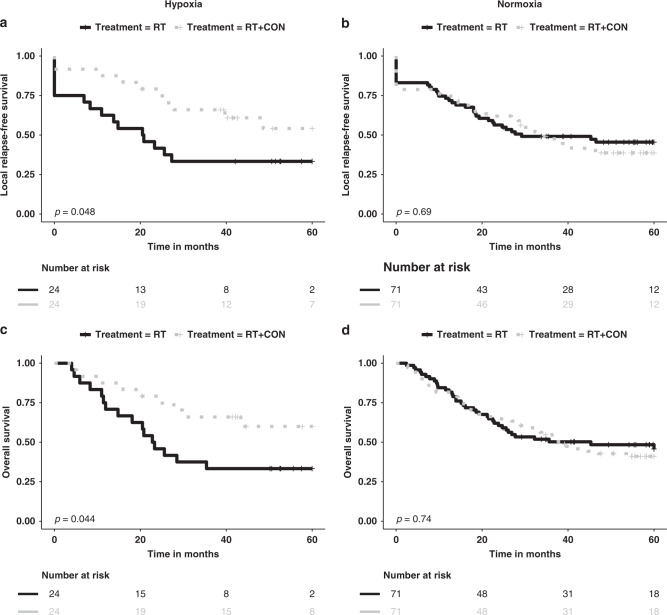
Performance of a 14-miRNA signature in the training and test datasets with stratification by the upper quartile of signature scores. Kaplan–Meier curves for local relapse-free (**a**, **b**) and overall (**c**, **d**) survival for patients with tumours classified as hypoxic and normoxic stratified by the upper quartile of the 14-miRNA signature score. BCON Bladder Carbogen and Nicotinamide, RT radiotherapy, CON carbogen–nicotinamide.

NanoString miRNA expression data were successfully generated for 190 out of 192 samples (95 RT and 95 RT+CON) available from patients in the BCON trial. The median cut-off in the BCON cohort did not predict benefit of having CON with RT for local relapse-free survival (LRFS; HR = 0.79, 95% CI 0.46–1.36, *p* = 0.40) and OS (HR = 0.77, 95% CI 0.45–1.34, *p* = 0.40). Using the upper quartile identified in the 62-patient cohort, the 14-miRNA signature predicted benefit of having CON with RT for LRFS (HR = 0.45, 95% CI 0.21–1.01, *p* = 0.048; Fig. 3a) and OS (HR = 0.44, 95% CI 0.19–1.00, *p* = 0.044; Fig. 3c) for patients classified as hypoxic. The benefit from CON was not significant in multivariable analyses for the patients classified as hypoxic for LRFS (HR = 0.53, 95% CI 0.24–1.19, *p* = 0.13) and OS (HR = 0.54, 95% CI 0.23–1.25, *p* = 0.15) (Supplementary Table 8). In patients classified as normoxic, there was no benefit from having CON (Fig. 3b, d). The interaction between the treatment arms and hypoxia status was borderline significant for LRFS (*p* = 0.06) and OS (*p* = 0.08). None of the individual miRNAs predicted benefit from CON (Supplementary Table 9).

The signature was not prognostic in the individual treatment arms of BCON for LRFS and OS (Supplementary Fig. 2). There were no statistically significant associations of the miRNA hypoxia/normoxia categories with clinico-pathologic variables except that hypoxic tumours had higher protein expression of CAIX (*p* = 0.0043) and HIF-1α (*p* = 0.015; Supplementary Table 10).

### Predicted targets of the miRNAs in the signature

mRNA target prediction for the 14 miRNAs in the signature identified 676 protein-coding genes. Reactome pathway analysis of the predicted miRNA targets showed that the top 10 most enriched pathways involved phosphoinositide-3 kinase (PI3K)/Akt signalling, regulation of transcription of phosphatase and tensin homologue (PTEN) and RUNX1 and oncogene-induced cell senescence (Table 2). Other enriched Reactome pathways (FDR < 0.05) were ‘Oxidative Stress Induced Senescence’, ‘VEGFA-VEGFR2 Pathway’, ‘Cyclin D-associated events in G1’ and ‘MAPK6/MAPK4 signalling’.

**Table 2:** Top 10 most enriched Reactome pathways for miRNA target protein-coding genes.

Reactome pathways	No. of genes	FDR
PI5P, PP2A and IER3 regulate PI3K/AKT signalling	17	0
PIP3 activates AKT signalling	15	0
Constitutive signalling by aberrant PI3K in cancer	14	0
Circadian clock	12	0
Oncogene-induced senescence	9	0
PI3K events in ERBB2 signalling	6	0.0008
Activated NTRK2 signals through FRS2 and FRS3	6	0.0008
Regulation of RUNX1 expression and activity	6	0.0008
Regulation of PTEN gene transcription	13	0.0008
EPHA-mediated growth cone collapse	9	0.0008

*FDR* false discovery rate.

### Performance of miRNA signatures in NMIBC

We had access to a miRNA profiled cohort for bladder cancer patients (*n* = 106), with most having NMIBC (*n* = 79). Expression profiling for one of the miRNAs in the signature (miR-224-5p) was not available and the signature score was calculated with the remaining 13 miRNAs. Values for miR-21-5p were missing for one of the NMIBC patients. Hence, 105 of the patients were used for the analyses. The signature was not prognostic for OS when stratified by the median (HR = 1.16, 95% CI 0.63–2.15, *p* = 0.63) or the upper quartile (HR = 1.33, 95% CI 0.69–2.57, *p* = 0.39).

We also assessed the performance of the six-miRNA hypoxia-regulated NMIBC cell line signature. The signature was prognostic in the mainly NMIBC patient cohort (OS; HR = 2.26, 95% CI 1.19–4.30, *p* = 0.011) using 5 of the 6 miRNAs (no data available for miR-708-5p) when the signature was summarised by mean expression and patients stratified by the upper quartile.

### Comparing the miRNA signature with the Yang mRNA signature

**Fig. 4:**
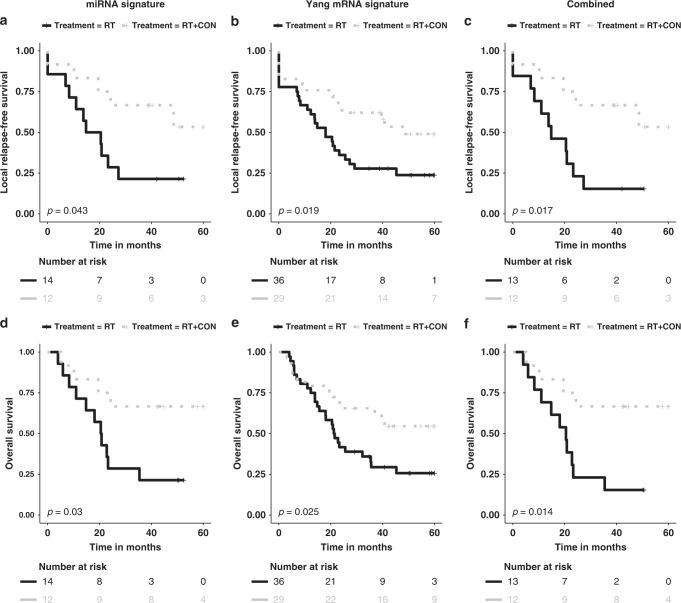
Combining miRNA and mRNA signatures improves performance. Kaplan–Meier curves for local relapse-free survival for patients with tumours classified as hypoxic using the miRNA signature (**a**), the Yang mRNA signature (**b**) and both signatures (**c**). Kaplan–Meier curves for overall survival for patients with tumours classified as hypoxic using the miRNA signature (**d**), the Yang mRNA signature (**e**) and both signatures (**f**). BCON Bladder Carbogen and Nicotinamide, RT radiotherapy, CON carbogen–nicotinamide.

Figure 4 shows the results of analyses of the 130 patients in BCON with both miRNA and mRNA signature scores. The miRNA signature predicted benefit from having CON for LRFS (HR = 0.35, 95% CI 0.12–1.02, *p* = 0.043) and OS (HR = 0.30, 95% CI 0.094–0.95, *p* = 0.030) in the 26 of the 130 patients it classified as hypoxic. The Yang mRNA signature performed similarly in predicting benefit from having CON for LRFS (HR = 0.47, 95% CI 0.25–0.91, *p* = 0.019) and OS (HR = 0.47, 95% CI 0.24–0.92, *p* = 0.025) in 65 of the 130 patients it classified as hypoxic. For the 25 patients classified as hypoxic by both signatures, the predictive benefit (based on the lower HRs) from having CON was higher for LRFS (HR = 0.29, 95% CI 0.097–0.85, *p* = 0.017) and OS (HR = 0.26, 95% CI 0.08–0.82, *p* = 0.014). The interaction between the hypoxia status and treatment arms was significant for LRFS (*p* = 0.033) and borderline significant for OS (*p* = 0.065). When categorising based on both signatures, there was a highly significant association with CA9 (*p* = 0.0008) and HIF-1α (*p* = 0.007) protein expression.

## Discussion

A hypoxia-associated miRNA signature was derived that predicted benefit from CON in patients with MIBC recruited in the Phase 3 BCON trial with trends for positive tests for interactions. The signature was associated with the protein expression of CAIX and HIF-1α confirming its hypoxia relevance. The miRNA did not outperform our mRNA signature, but a combination was superior. Combining the miRNA signature with our previously derived 24-gene mRNA classifier identified a small group of patients (25/130) who derived considerable benefit from hypoxia-modifying treatment.

The ability of the miRNA signature to predict benefit from hypoxia-modifying CON is consistent with our previous work with mRNA signatures in not only bladder cancer^9^ but also laryngeal tumours.^7^ Together these findings provide evidence that the most hypoxic tumours are most responsive to CON across tumour types. It was not feasible to compare the performance of the miRNA hypoxia signature with CAIX and HIF-1α given protein expression data were available for only 84 patients with miRNA expression data. However, CAIX and HIF-1 α have been shown previously to predict benefit from CON in BCON in a larger cohort of 138 and 137 patients, respectively.^8^

Different approaches are used for generating signatures associated with tumour hypoxia. Here we combined a widely used seed gene method with the one used previously to identify hypoxia-related miRNAs by associating with mRNA classifications.^36,37^ Although miRNAs can have a tissue-specific response to hypoxia, some miRNAs are consistently regulated by hypoxia across tumour types.^40^ Nine of the 62 miRNA seed genes used to develop our bladder signature were among the 23 miRNAs identified as hypoxia regulated in breast and colon cancer cell lines.^41^ All 14 miRNAs in the signature had been studied in relation to hypoxia with 12 induced in cancer cell lines,^18,42–46^ 1 (miR-190a-5p) in pulmonary endothelial cells^47^ and miR-28-5p was downregulated in mice PC-12 cells.^48^ miR-210-3p is the most widely known hypoxia-associated miRNA; it is both induced by and stimulates the stabilisation of HIF-1α.^49^ miR-210-3p regulates the cell cycle, DNA repair, metabolism and angiogenesis during hypoxia.^49^ miR-210-3p was an independent prognostic factor in breast, head and neck and colorectal cancers but not in bladder cancer.^50–53^ miR-21-5p is another prominent hypoxia-associated oncogenic miRNA in the signature^54^ that is associated with a poor prognosis in bladder cancer.^55^ miR-193b-3p is in a six-miRNA NMIBC hypoxia signature.^18^

KEGG pathways associated with the predicted targets of the 14 miRNAs showed that PI3K/Akt signalling was one of the most significantly enriched. Of note, HIF-1 is a protein downstream of the PI3K/Akt signalling pathway.^56^ Regulation of *PTEN* gene transcription was also enriched, and *PTEN* loss promotes HIF-1 transcriptional activity.^57^ Under hypoxia, RUNX1 interacts with HIF-1α stimulating the expression of RUNX1-regulated genes contributing to hematopoietic stem cell differentiation.^58^ The pathway analysis, therefore, adds further support for the hypoxia relevance of our miRNA signature.

The miRNA hypoxia signature was not prognostic in the mainly NMIBC patient bladder cancer cohort. Given the differences in molecular alterations between the two subtypes of bladder cancer and involvement of different miRNAs in both subtypes, a MIBC miRNA signature may not perform in a mainly NMIBC cohort.^59,60^ The NMIBC signature showed mixed performance in MIBC cohorts as it was prognostic in the mainly MIBC TCGA BLCA cohort but was not predictive or prognostic in the RT arm of BCON or the 62-patient MIBC cohort.

The signature outperformed use of a single miRNA in its ability to predict benefit from CON highlighting the importance of developing signatures rather than individual genes as biomarkers. However, the signature did not outperform our 24-gene mRNA signature possibly because it had fewer genes. Combining the two signatures identified the patients who were the most hypoxic. The combined power of mRNA–miRNA signatures has been shown previously. A combined mRNA–miRNA leucocyte-associated signature was more prognostic than the individual signatures for survival in TCGA ovarian cancer cohort.^61^

A limitation of our study was the mixed performance of the signature in surgically treated MIBC despite tumour hypoxia being associated with a poor prognosis in cancer patients whether treated by primary surgery or RT.^9^ Another limitation of the miRNA hypoxia signature was that the initial predetermined median cut-off identified in the TCGA training cohort did not perform consistently, and hence we changed to using an upper quartile, which was identified in the 62-patient MIBC cohort. Finding cut-offs that are robust across a range of data sets with expression profiling using a range of different technologies was a challenge. This challenge could be addressed if more data sets were available particularly those involving RT. In summary, a 14-miRNA hypoxia signature was developed that predicted benefit from having hypoxia modifying with RT for patients with MIBC. Combining the miRNA with our 24-gene mRNA signature identified a small group of patients who derived considerable benefit from hypoxia modification. Future work should focus on combining hypoxia classifications from both signatures to identify patients with the most hypoxic tumours. The next step to validate the miRNA hypoxia signature is to test its performance in combination with the mRNA hypoxia signature in a prospective clinical trial.

## Supplementary information

Supplementary information

## Data Availability

The mRNA (GSE48075), miRNA (GSE84525) expression and clinical (GSE84525) data are publicly available for the 62 MIBC patients. The BCON NanoString miRNA data are available upon request. miRNA expression data for the 106 bladder cancer patients are available on request.
