# Supplementary material for: A miRNA signature predicts benefit from addition of hypoxia-modifying therapy to radiation treatment in invasive bladder cancer
Source: Br J Cancer. 2021 Apr 12;125(1):85–93. doi: 10.1038/s41416-021-01326-9 (PMC8257670; doi:10.1038/s41416-021-01326-9)
Supplement: Supplementary file 1 — Supplementary information [file 41416_2021_1326_MOESM1_ESM.docx]

**Supplementary Information**

**Supplementary methods**

Boruta offers an improved method for computing feature importance compared to using random forest as random forest importance measures are subject to variability every time the random forest algorithm is run. Briefly the Boruta algorithm created a dataset with all the features (miRNAs) and shuffled copies of each of the features, known as shadow features. The extended dataset consisting of the features and shadow features was then analysed using a random forest algorithm. The random forest computed the mean decrease in accuracy for each of the features and shadow features across all trees in the random forest after using shuffled values for each of them. A Z-score (importance) was computed for each feature as the mean decrease in accuracy divided by its standard deviation. The Z-scores for each feature were compared with the maximum Z-scores among the shadow features and the miRNAs with Z-scores significantly higher (p<0.01, binomial test) than the maximum Z-score among the shadow features were computed as important for classification. The miRNAs with Z-scores significantly lower than the maximum Z-score among the shadow features were classified as unimportant for classification and removed from the dataset. This process was repeated for 1,000 iterations, with new shadow features created at each iteration.

In order to determine the Gini importance of the miRNAs in the signature, a random forest model was built using all the miRNA seed genes and the R package randomForest (v 4.6) ^62^. The default number of trees was 500 and number of variables for splitting each tree (mtry value) was the square root of the number of miRNAs.

RNA was extracted for the BCON samples using three 20 µM sections of the formalin-fixed paraffin-embedded (FFPE) tissue blocks and the RecoverAll Total Nucleic Acid Isolation Kit (Thermo Fisher Scientific). The median of the 260/280 ratio for the samples was 1.96, with only 11 samples having 260/280 ratios <1.5. The NanoString protocol was carried out with 12 samples per run as per the manufacturer’s instructions at the Genomic Technology Core Facility, University of Manchester. The RCC files were loaded and processed in nSolver version 4.0. From the raw miRNA counts for each of the samples, positive control normalisation was undertaken in which the counts were normalised by multiplying with a positive control normalisation factor, which was calculated as the geometric mean of the positive controls across all samples over the geometric mean of the positive controls in the given sample. The counts were then normalised by multiplying with the housekeeping normalisation factor, which was calculated as the geometric mean of the top 100 most expressed miRNAs across all samples over the geometric mean of the top 100 most expressed miRNAs in the given sample. Two samples were removed, one had a quality control flag in the ligation quality control, while the other had the positive normalisation factor outside the acceptable range. A ligation control quality control flag can indicate the presence of chaotropic salts in the sample. The accepted range for the positive control normalisation factor is 0.3-3.0, a factor outside the range may indicate a high amount of degradation in the sample. The log_2_ transformed normalised counts of the 190 samples without flags in the BCON cohort were used for the analysis.

62. Breiman, L. randomForest: Breiman and Cutler’s random forests for classification and regression. Version 4.6 (2018).

Supplementary Fig. 1. miRNA and Yang mRNA signatures were prognostic for overall survival in the 62 muscle invasive bladder cancer cohort. Kaplan-Meier curves for overall survival for miRNA (A) and Yang mRNA (B) signatures. Kaplan Maier curves for disease specific survival for miRNA (C) and Yang mRNA (D) signatures. The miRNA signature and the Yang mRNA signature were stratified by the upper and lower quartile respectively.

B

A

D

C

Supplementary Fig. 2. The 14-miRNA hypoxia signature was not prognostic in BCON.

Kaplan-Meier curves for local relapse free survival in the RT (A) and RT+CON (B) arms of BCON. Kaplan Maier curves for overall survival in the RT (C) and RT+CON (D) arms of BCON.

BCON: Bladder Carbogen and Nicotinamide; RT: radiotherapy; CON: carbogen-nicotinamide.

B

A

C

D

##### Supplementary Table 1. Patient characteristics for the TCGA BLCA training and test datasets

| Variable |  | Training (n=284) | Test (n=121) | p |
| --- | --- | --- | --- | --- |
| Gender | Male | 208 (73.3%) | 90 (74.4%) |  |
|  | Female | 75 (26.4%) | 31 (25.6%) | 0.95^1^ |
| Median (range) age (years) |  | 69 (34-90) | 69 (42-89) | 0.34^2^ |
| Stage | TX/T0 | 2 (0.7%) | 0 (0%) |  |
|  | T1 | 1 (0.4%) | 2 (1.7%) |  |
|  | T2 | 84 (29.6%) | 34 (28.1%) |  |
|  | T3 | 133 (46.8%) | 59 (48.8%) |  |
|  | T4 | 39 (13.7%) | 18 (14.9%) | 0.56^1^ |
|  | No Data | 25 (8.8%) | 8 (6.6%) |  |

^1^Chi-Sq test

^2^Unpaired t-test with Welch correction

TCGA BLCA: The Cancer Genome Atlas bladder cancer

##### Supplementary Table 2. Patient characteristics for the BCON miRNA cohort by randomisation arm

| Variable |  | RT (n=95) | RT+CON (n=95) | p |
| --- | --- | --- | --- | --- |
| Gender | Male | 74 (77.9%) | 78 (82.1 %) |  |
|  | Female | 21 (22.1%) | 17 (17.9%) | 0.59^1^ |
| Median (range) age (years) | | 75.5 (51.1-88.9) | 74.8 (51.5-89.7) | 0.38^2^ |
| Stage | T1 | 1 (1.0 %) | 9 (9.5%) |  |
|  | T2 | 60 (63.2%) | 69 (72.6%) |  |
|  | T3 | 22 (23.2%) | 14 (14.7%) |  |
|  | T4 | 4 (4.2 %) | 3 (3.2%) | 0.47^1^ |
| Grade | 3 | 95 (100%) | 95 (100%) |  |
| Concurrent pTis | Absent | 60 (63.2%) | 79 (83.2%) |  |
|  | Present | 35 (36.8%) | 16 (16.8%) | 0.003^1^ |
| TURBT | Biopsy | 23 (24.2%) | 24 (25.3%) |  |
|  | Partial | 29 (30.5%) | 31 (32.6%) |  |
|  | Complete | 39 (41.1%) | 37 (38.9%) | 0.93^1^ |
|  | No data | 4 (4.2%) | 3 (3.2%) |  |
| Necrosis | Absent | 46 (48.4%) | 43 (45.3%) |  |
|  | Present | 49 (51.6%) | 52 (54.7%) | 0.77^1^ |

^1^Chi-Sq test

^2^Unpaired t-test with Welch correction

BCON: Bladder Carbogen and Nicotinamide; Concurrent pTis: Carcinoma in situ; TURBT: transurethral resection of bladder tumour

##### Supplementary Table 3. Fewer miRNAs induced under 1% O_2_ versus 0.2% O_2_ in Taqman array A cards

| Cell lines^1^ | 0.2% array A | 1% array A |
| --- | --- | --- |
| T24 | 64 | 10 |
| J82 | 46 | 18 |
| UMUC3 | 60 | 10 |
| RT4 | 41 | - |

^1^Array A cards only run at 0.2% O_2_ for RT4 cell line

#####

#####

##### Supplementary Table 4. Seed genes used to develop a miRNA hypoxia signature

| Seed genes | |
| --- | --- |
| let-7a-5p | miR-22-3p |
| let-7c-5p | miR-221-3p |
| let-7d-5p | miR-454-3p |
| let-7e-5p | miR-224-5p |
| let-7f-5p | miR-26a-5p |
| miR-100-5p | miR-27a-3p |
| miR-103a-3p | miR-27b-3p |
| miR-210-3p | miR-28-5p |
| miR-130a-3p | miR-29a-3p |
| miR-125a-5p | miR-301b-3p |
| miR-125b-5p | miR-30b-5p |
| miR-125a-3p | miR-339-5p |
| miR-128-3p | miR-320a |
| miR-130b-3p | miR-324-3p |
| miR-146b-5p | miR-324-5p |
| miR-93-5p | miR-328-3p |
| miR-19a-3p | miR-345-5p |
| miR-15b-5p | miR-361-5p |
| miR-190a-5p | miR-365a-3p |
| miR-183-5p | miR-423-5p |
| miR-181a-5p | miR-425-5p |
| miR-182-5p | miR-455-5p |
| miR-18a-5p | miR-483-5p |
| miR-18b-5p | miR-484 |
| miR-191-5p | miR-574-3p |
| miR-193a-5p | miR-579-3p |
| miR-193b-3p | miR-597-5p |
| miR-19b-3p | miR-92a-3p |
| miR-20a-5p | miR-671-3p |
| miR-20b-5p | miR-99b-5p |
| miR-21-5p | miR-491-5p |

##### Supplementary Table 5. Univariable and multivariable analyses for the TCGA BLCA training dataset

|  | Univariable | | Multivariable | |
| --- | --- | --- | --- | --- |
| Overall survival | **HR [95% CI]** | **p** | **HR [95% CI]** | **p** |
| Stage (T3-4) | **2.49[1.09-5.67]** | **0.02** | **2.38 [1.04-5.43]** | **0.039** |
| Age (continuous) | **1.03[1.01-1.05]** | **0.0007** | **1.03 [1.01-1.05]** | **0.0022** |
| Male | 0.87[0.59-1.29] | 0.50 |  |  |
| Hypoxia | **1.41 [0.99-2.02]** | **0.056** | **1.41 [0.97-2.05]** | **0.071** |
| Progression free survival | **HR [95% CI]** | **p** | **HR [95% CI]** | **p** |
| Stage (T3-4) | **2.26 [1.05-4.86]** | **0.03** | **2.15 [1.00 -4.62]** | **0.051** |
| Age (continuous) | 1.01[1.0-1.03] | 0.10 |  |  |
| Male | 0.81[0.55-1.19] | 0.30 |  |  |
| Hypoxia | **1.71 [1.20-2.45]** | **0.0027** | **1.72 [1.17- 2.51]** | **0.0053** |

TCGA BLCA: The Cancer Genome Atlas bladder cancer

#####

##### Supplementary Table 6. Univariable and multivariable analyses for the TCGA BLCA test dataset

|  | Univariable | | Multivariable | |
| --- | --- | --- | --- | --- |
| Overall survival | **HR [95% CI]** | **p** | **HR [95% CI]** | **p** |
| Stage (T3-4) | 1.23[0.44-3.44] | 0.70 |  |  |
| Age (continuous) | **1.03[1.01-1.06]** | **0.02** | **1.03 [1.01-1.06]** | **0.019** |
| Male | 0.90 [0.47-1.72] | 0.80 |  |  |
| Hypoxia | **1.53 [0.84-2.76]** | **0.16** | **1.69 [0.91- 3.15]** | **0.096** |
| Progression-free survival | **HR [95% CI]** | **p** | **HR [95% CI]** | **p** |
| Stage (T3-4) | 0.97 [0.38-2.49] | 0.90 |  |  |
| Age (continuous) | 1.01[0.98-1.04] | 0.40 |  |  |
| Male | 1.35[0.64-2.82] | 0.40 |  |  |
| Hypoxia | 1.75 [0.94-3.27] | 0.074 |  |  |

TCGA BLCA: The Cancer Genome Atlas bladder cancer

##### Supplementary Table 7. Univariable and multivariable analyses in the 62 patient McConkey cohort

|  | Univariable | | Multivariable | |
| --- | --- | --- | --- | --- |
| Overall survival | **HR [95% CI]** | **p** | **HR [95% CI]** | **p** |
| Stage (T3-4) | 2.14 [0.99-4.62] | **0.05** | 1.95 [0.84-4.50] | **0.12** |
| N + | 4.07 [1.78-9.30] | **0.0009** | 5.80 [2.09-16.1] | **0.00073** |
| Hypoxia miRNA  (median) | 2.56 [1.19-5.48] | **0.01** | 1.11 [0.37-3.31] | **0.85** |
| Hypoxia miRNA  (upper quartile) | 3.69 [1.77-7.69] | **0.0002** | 2.01 [0.70-5.81] | **0.20** |
| Hypoxia Yang mRNA  (median) | 1.51 [0.73-3.11] | 0.26 |  |  |
| Hypoxia Yang mRNA  (lower quartile) | 3.21 [1.12-9.23] | **0.022** | 2.81 [0.84-9.43] | **0.09** |
| Age (continuous) | 1.07 [1.02-1.11] | **0.002** | 1.07 [1.02-1.13] | **0.0092** |
| Disease-specific survival | **HR [95% CI]** | **p** | **HR [95% CI]** | **p** |
| Stage (T3-4) | 2.32 [1.02-5.28] | **0.04** | 2.14 [0.88-5.21] | **0.094** |
| N + | 4.74 [2.04-11.0] | **0.0003** | 6.89 [2.38-19.9] | **0.00037** |
| Hypoxia miRNA  (median split) | 2.57 [1.15-5.73] | **0.02** | 0.93 [0.29-3.05] | **0.91** |
| Hypoxia miRNA  (upper quartile) | 3.87 [1.79-8.39] | **0.00022** | 2.32 [0.74-7.25] | **0.15** |
| Hypoxia Yang mRNA  (median) | 1.45 [0.68-3.10] | 0.34 |  |  |
| Hypoxia Yang mRNA  (lower quartile) | 2.86 [0.99-8.28] | **0.043** | 2.52 [0.74-8.61] | **0.14** |
| Age (continuous) | 1.05 [1.01-1.10] | **0.01** | 1.05 [1.00- 1.11] | **0.06** |

N+: Lymph node invasion; M+: distant metastasis

##### Supplementary Table 8. Univariable and multivariable analyses of clinico-pathological variables in 48 BCON patients with tumour hypoxia (high miRNA signature scores)

|  | Univariable | | Multivariable | |
| --- | --- | --- | --- | --- |
| Local relapse-free survival | **HR [95% CI]** | **p** | **HR [95% CI]** | **p** |
| Stage (T3-4) | 1.06 [0.47-2.37] | 0.90 |  |  |
| CIS present | 1.79 [0.82-3.92] | 0.10 |  |  |
| Necrosis present | 0.86 [0.39-1.91] | 0.70 |  |  |
| Age (continuous) | **1.10 [1.03-1.17]** | **0.002** | **1.09 [1.03-1.16]** | **0.004** |
| Male | 0.93 [0.35-2.48] | 0.90 |  |  |
| CON | **0.45 [0.21-1.01]** | **0.048** | **0.53 [0.24-1.19]** | **0.13** |
| Overall survival | **HR [95% CI]** | **p** | **HR [95% CI]** | **p** |
| Stage (T3-4) | 1.09 [0.47-2.52] | 0.80 |  |  |
| CIS present | 1.83 [0.83-4.04] | 0.10 |  |  |
| Necrosis present | 0.89 [0.40-1.98] | 0.80 |  |  |
| Age (continuous) | **1.09 [1.03-1.15]** | **0.005** | **1.08 [1.02-1.14]** | **0.012** |
| Male | 0.91 [0.34-2.41] | 0.80 |  |  |
| CON | **0.44 [0.19-1.00]** | **0.044** | **0.54 [0.23-1.25]** | **0.15** |

BCON: Bladder Carbogen and Nicotinamide; CIS: carcinoma in situ; CON: carbogen- nicotinamide

**Supplementary Table 9. None of the individual miRNAs predicted benefit from CON**

| Overall survival  miRNA high expression^1^ | HR [95% CI]5% CI] | p |
| --- | --- | --- |
| miR-27a-3p | 0.96 [0.44-2.06] | 0.91 |
| miR-193b-3p | 0.58 [0.26-1.28] | 0.17 |
| miR-455-5p | 0.49 [0.21-1.18] | 0.11 |
| miR-221-3p | 0.57 [0.27-1.23] | 0.15 |
| miR-210-3p | 0.74 [0.35-1.55] | 0.42 |
| miR-21-5p | 0.56 [0.26-1.20] | 0.13 |
| miR-224-5p | 1.10 [0.46-2.62] | 0.82 |
| miR-491-5p | 0.98 [0.44-2.16] | 0.96 |
| miR-93-5p | 0.67 [0.30-1.52] | 0.34 |
| miR-182-5p | 0.91 [0.43-1.93] | 0.81 |
| miR-30b-5p | 0.59 [0.27-1.28] | 0.18 |
| miR-190a-5p | 1.14 [0.52-2.50] | 0.74 |
| miR-28-5p | 1.03 [0.47-2.27] | 0.93 |
| miR-191-5p | 0.94 [0.44-2.00] | 0.87 |

^1^miRNA expression stratified by upper quartile

##### Supplementary Table 10. BCON patient clinico-pathological variables stratified by hypoxia signature

| Variable |  | Hypoxic (n=48) | Normoxic (n=142) | p |
| --- | --- | --- | --- | --- |
| Treatment | RT  RT+CON | 26 (54.2 %)  22 (45.8 %) | 69 (48.6%)  73 (51.4 %) | 0.62^1^ |
| Median (range) age (years) |  | 75.7 [51.5-88.9] | 74.8 [51.1-89.7] | 0.44^2^ |
| Tumour stage | T1-T2  T3-T4a | 32 (66.7%)  16 (33.3%) | 115 (81.0%)  27 (19.0%) | 0.06^1^ |
| TURBT | Complete  Partial/Biopsy | 17 (35.4%)  29 (60.4%) | 78 (55.0%)  59 (42.0%) | 0.58 ^1^ |
| Gender | Male  Female | 39 (81.3 %)  9 (18.8%) | 113 (79.6 %)  29 (20.4 %) | 0.97^1^ |
| Necrosis | Present  Absent | 31(64.6 %)  17 (35.4 %) | 70 (49.3 %)  72 (50.7 %) | 0.095^1^ |
| CIS | Present  Absent | 17 (35.4%)  31 (64.6 %) | 34 (23.9 %)  108 (76.1%) | 0.17^1^ |
| CAIX^4^ | Median [Range]  Data available | 21 [0-173.8]  31 [64.6 %] | 1.65 [0-173.3]  78 [54.9%] | 0.0043^3^ |
| Glut-1^4^ | Median [Range]  Data available | 126.3 [3.4-275.8]  23 [47.9%] | 82.5 [0-300]  57 [40.1 %] | 0.26^3^ |
| HIF-1α^4^ | Median [Range]  Data available | 35.0 [0.80-140.4]  23 [47.9 %] | 16.8 [0-169.4]  61 [43.0 %] | 0.015^3^ |

^1^Chi-sq

^2^unpaired t-test with Welch correction

^3^Mann-Whitney U test

^4^H-scores

BCON: Bladder Carbogen and Nicotinamide; TURBT: transurethral resection of bladder tumour; CIS: carcinoma in situ; CAIX: carbonic anhydrase IX; Glut-1: glucose transporter-1; HIF-1α: hypoxia inducible factor 1 alpha
